# Telepsychiatry care during the COVID-19 outbreak in young adults with a first episode of psychosis or entering schizophrenia

**DOI:** 10.1192/j.eurpsy.2021.932

**Published:** 2021-08-13

**Authors:** E. Kiesmann, J. Mallet, V. Charlot, C. Dubertret

**Affiliations:** Psychiatry, AP-HP, Louis Mourier Hospital, Colombes, Université de Paris, Faculté de médecine. INSERM UMR1266, Institute of Psychiatry and Neuroscience of Paris, France, COLOMBES, France

**Keywords:** Covid-19, telepsychiatry, first episode of psychosis, schizophrenia

## Abstract

**Introduction:**

During the Covid Outbreak, the deployment of psychiatric phone-based consultations (PbC) became a large necessity.

**Objectives:**

The main objective of our study was to assess, 4 months after the end of the lockdown, the degree of satisfaction of the PbCs compared to that of usual face-to-face consultations (FC) in young adults presenting a first episode of psychosis (FEP) or entering schizophrenia (SCZ).

**Methods:**

All patients beneficiated from PbCs conducted by hospital care staff during lockdown. A 15-items questionnaire evaluating satisfaction was carried out remotely (score ranging from 1 to 10). Primary outcome was satisfaction with consultation allowing the comparison of a group preferring FC (FC+) against a group in favor or equivalent of PbC (PbC +).

**Results:**

30 patients were recruited (mean age 26.93 years old (4.9 SD), Male 56%. Diagnoses were SCZ 60% and FEP 40%. 20/30 participants belonged to (FC+) group. Total scores of satisfaction for the PbC differed between the (PbC+) group (mean 9 (1.69 SD)) and (FC+) group (mean 6.80, (1.32 SD)) p < 0.05. The (FC+) group tends to have PbC more frequently (40%) than the (PbC+) group (10%) and to find the phone interface more stressfull (40%) than the PbC+ group (10%). The (FC+) group tends to less wish (40%) PbC follow up in future than the (PbC+) group (90%).

**Conclusions:**

This study shows that the PbCs were favorably evaluated by a third of the patients. The anxiety-inducing experience of the PbC in the (FC+) group could be explained by the severity of their pathology.

